# Chiral Plasmonics and Their Potential for Point-of-Care Biosensing Applications

**DOI:** 10.3390/s20030944

**Published:** 2020-02-10

**Authors:** Willian A. Paiva-Marques, Faustino Reyes Gómez, Osvaldo N. Oliveira, J. Ricardo Mejía-Salazar

**Affiliations:** 1 National Institute of Telecommunications (Inatel), Santa Rita do Sapucaí MG 37540-000, Brazil; willianmarques@geb.inatel.br; 2 Sao Carlos Institute of Physics, University of Sao Paulo, PO Box 369, Sao Carlos 13560-970, SP, Brazil; faustino.reyes@correounivalle.edu.co (F.R.G.);

**Keywords:** plasmonics, biosensing, telecommunications

## Abstract

There has been growing interest in using strong field enhancement and light localization in plasmonic nanostructures to control the polarization properties of light. Various experimental techniques are now used to fabricate twisted metallic nanoparticles and metasurfaces, where strongly enhanced chiral near-fields are used to intensify circular dichroism (CD) signals. In this review, state-of-the-art strategies to develop such chiral plasmonic nanoparticles and metasurfaces are summarized, with emphasis on the most recent trends for the design and development of functionalizable surfaces. The major objective is to perform enantiomer selection which is relevant in pharmaceutical applications and for biosensing. Enhanced sensing capabilities are key for the design and manufacture of lab-on-a-chip devices, commonly named point-of-care biosensing devices, which are promising for next-generation healthcare systems.

## 1. Introduction

Objects whose mirror images cannot be superimposed are named chiral, with our hands being the most universal example. This geometrical handedness, called chirality, is ubiquitous in nature and plays a key role in the chemical and biological activity of molecules. Opposite mirror images of a chiral molecule that share the same stoichiometric molecular formula are known as enantiomers, being divided into left- (L) and right-handedness (R), respectively. Discrimination of enantiomers is critical in biology and pharmaceutics, where L and R enantiomers may be two separate drugs with different metabolic profiles and affinities for receptors or enzymes [1,2]. The therapeutic effects of a chiral drug are often associated with a single enantiomer whereas the other is inactive and/or contributes to undesirable effects [1,2,3,4,5,6]. Thalidomide is one of the most notorious examples, as its L enantiomer produced the teratogenic side effects responsible for limb malformations in the late 1950s [7,8,9]. Furthermore, toxicity due to proteins with wrong chirality is believed to be one of the underlying causes of diseases such as Alzheimer’s, Parkinson’s, Huntington’s, and type II diabetes [10]. Since opposite enantiomers are identical in their chemical composition and scalar physical properties, separation techniques must rely on their interaction with other chiral objects. Circularly polarized light (CPL), divided into left- (LCP) and right-hand polarizations (RCP), is routinely used to discriminate molecular chirality as an alternative to the use of other chiral samples that may introduce unwanted side products. However, due to the mismatch between the molecules sizes and the wavelength of the incident CPL, chiral molecules typically exhibit very weak circular dichroism (CD), of the order of tens of millidegrees in the ultraviolet region [11], restricting its application to large sample volumes or large molecules.

Plasmonic platforms, with the unique ability to confine and manipulate light through the resonant coupling of light to collective oscillations of free electrons in metals, are promising for enhancing the chiroptical response [12,13,14,15,16,17,18,19,20,21,22,23,24]. In particular, geometrically chiral plasmonic structures have been used to tailor and tune the far- and near-field chiroptical responses [25,26,27,28,29,30,31,32,33,34,35,36,37]. The interaction of plasmonic enhanced chiral near-field with a chiral molecule is favorable to optimize the selective light absorption by the molecule, which in turn optimizes the corresponding CD signal [38,39,40,41,42]. We review here the recent trends in chiral plasmonic systems for enantioselective probing of biomolecules, with emphasis on the limitations of the systems reported so far. In addition, an outlook is presented of the application of chiral plasmonics for point-of-care biosensing applications.

## 2. Plasmonics for Enhanced Chiroptical Effects

The electric, $\tilde{p}$, and magnetic, $\tilde{m}$, dipolar moments, generated by a chiral molecule upon illumination with a monochromatic, time-harmonic and low-intensity electromagnetic field, are described by

(1) $$\begin{array}{c} \tilde{p}=\tilde{\alpha }Ẽ-i\tilde{G}\tilde{B}, \end{array}$$

(2) $$\begin{array}{c} \tilde{m}=\tilde{\chi }\tilde{B}+i\tilde{G}Ẽ, \end{array}$$

where $\tilde{\alpha }$, $\tilde{\chi }$, and $\tilde{G}$, are the complex electric polarizability, magnetic susceptibility and the mixed electric-magnetic dipole polarizability, respectively. **Ẽ** and $\tilde{B}$ are the complex local electric and magnetic fields at the molecule [43]. The absorption of light by the molecule can be described by [44]

(3) $$A^{\pm }=\langle E\cdot p+B\cdot m\rangle =\frac{\omega }{2}\left(\alpha^{\prime \prime }{\left|Ẽ\right|}^{2}+\chi^{\prime \prime }{\left|\tilde{B}\right|}^{2}\right)\mp \frac{2}{\varepsilon_{0}}G^{\prime \prime }C,$$

for RCP (+) and LCP (−) polarizations. The symbol ” stands for the imaginary part of complex quantities and *C* denotes the chiral density - the degree of chiral asymmetry in the absorption of a molecule - defined by [45]

(4) C=ε02E·∇×E+12μ0B·∇×B=−ωε02ImE˜∗·B˜,

where $\varepsilon_{0}$ and $\mu_{0}$ are the permittivity and permeability of free space, respectively. From Equation (3) one notes that $CD∼\left(A^{+}-A^{-}\right)=-\frac{4}{\varepsilon_{0}}G^{\prime \prime }C$, the differential absorption of light depends on the chirality of matter ($G^{\prime \prime }$) and the chirality of the local electromagnetic field (*C*). The chiral density of CPL in a vacuum reaches the maximum value $C_{\mathrm{CPL}}^{\pm }=\pm \frac{\omega \varepsilon_{0}}{2c}{\left|E\right|}^{2}$ [19], where E is the electric field amplitude of the incident CPL. Therefore, engineering resonant structures with localized, enhanced electromagnetic near-fields will produce spatially averaged enhancements of C, which in turn enhances the corresponding CD signal, CD/CD${}_{\mathrm{CPL}}$ = $C/C_{\mathrm{CPL}}=\hat{C}$. It is in this context that the unique ability of plasmonics can be successfully exploited. In this section, we will discuss some of the ways to design and develop chiral plasmonic structures to tailor and tune the localization and enhancement of chiral near-fields. The most common design philosophy uses the spatial symmetry breaking to control the near-field coupling of the elements of the nanostructures [19].

**Figure 1:**
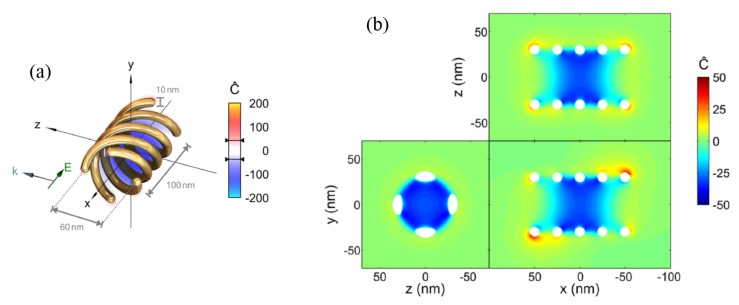
(**a**) Schematics of a plasmonic helix with a pitch of 100 nm and a diameter of 60 nm for the excitation of enhanced chiral near-fields. Due to the chiral geometry of the helix, the chiral density can be excited even from incident linearly polarized fields, as depicted. (**b**) Slice plots confirming the enhanced chiral density in the interior region of the helix due to the excitation of plasmonic resonances. The results shown are for an incident wavelength λ=1630 nm. Reproduced with permission from ref. [19]. Copyright 2014 American Chemical Society.

**Figure 2:**
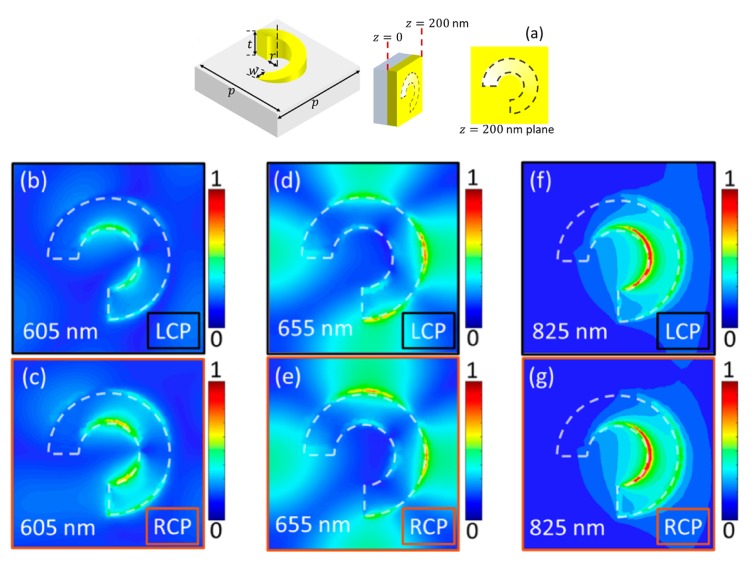
(**a**) Illustration of a single ramp-shaped chiral plasmonic nanostructure and its geometrical parameters. (**b**)–(**g**) The near-field maps at the three major resonances of the structure: 605 nm; 655 nm; and 825 nm for LCP and RCP incident light. Calculations were performed for a 2D periodic array of ramp-shaped structures, with r=w=100 nm, t=200 nm and a period length of p=600 nm. Results for the electric field amplitude are normalized to $E_{0}=1.76\times 10^{9}$. Reproduced with permission from ref. [49]. Copyright 2019 American Chemical Society.

Chiral plasmonic nanostructures are advantageous due to the strongly localized electromagnetic fields, with which they can support chiral eigenmodes, i.e., the near-fields of the eigenmodes are chiral [19]. Importantly, these eigenmodes can be excited by achiral linearly polarized plane waves, as depicted in Figure 1a, which may reduce the parameters to be controlled for experimental reliable designs. The near-field coupling of neighboring scatterers produces a collective chiral behavior, with high optical chirality over an extended region inside the helical structure, as shown in Figure 1b. Despite the advantages of this type of chiral plasmonic structure, setting nanoparticles at customized regions for high chiral interactions constitutes a major hurdle for practical biosensing applications. A recent approach to circumvent this latter issue is the use of plasmonic chiral metasurfaces [46,47,48,49,50,51,52,53,54], where several 2D plasmonic arrays with symmetry broken along the third dimension have been developed. These metasurfaces have their chiral plasmonic near-fields directly accessible to nearby molecules on the surface. Figure 2a shows the schematics of a ramp-shaped plasmonic nanostructure. Metasurfaces comprised of a 2D periodic array of these gradient depth unit-cells were produced in Reference [49] using the focused ion beam (FIB) method. Plasmonic resonances were characterized for these nanostructures under LCP and RCP incident light, with high dissymmetries in the reflection spectra at the incident wavelengths λ=605 nm and λ=655 nm. This chiroptical effect was explained in terms of the asymmetry in the corresponding near-fields for both polarizations, as clearly noted from Figure 2b–e. For λ=825 nm no differences were observed in the reflection spectra for RCP and LCP, as expected from the near-field maps in Figure 2f–g.

**Figure 3:**
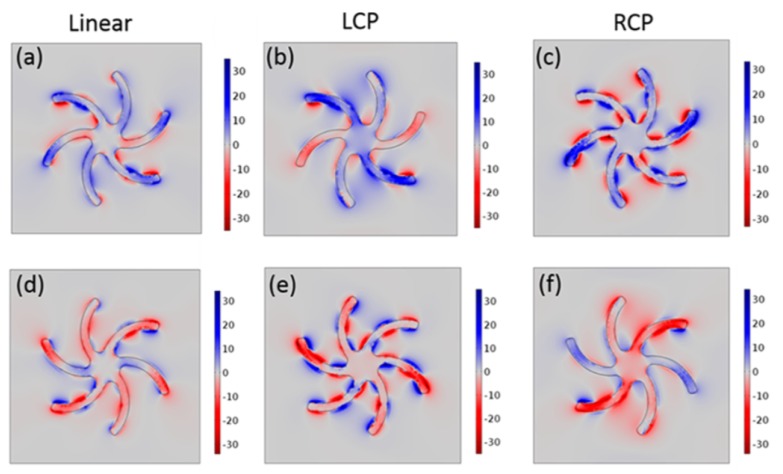
Calculated chiral density near-field maps for a shuriken-shaped structure in water for (**a**)–(**c**) left-handed (LH) and (**d**)–(**f**) right-handed (RH) geometries. Results are presented for LCP, RCP, and linearly polarized incident light. Reproduced with permission from ref. [57]. Copyright 2018 American Chemical Society.

The main limitation of a geometry-focused approach for chiral plasmonics is that the functionality cannot be easily manipulated post-fabrication. This drawback has motivated research work to fabricate plasmonic nanostructures whose chiroptical properties can be manipulated [55,56,57,58,59,60,61,62,63]. Figure 3 shows the calculated near-field chiral density ($\hat{C}$) for a shuriken-shaped nanostructure [57]. Calculations were performed to show the corresponding chiral electromagnetic near-fields under linearly and circularly polarized incident light. The high sensitivity of plasmonic resonances to small changes in the properties of the surrounding dielectric media can also be exploited for external control of these chiral density maps [57]. Although this mechanism for the control of chiroptical interaction can open new avenues for applications, the need to use chiral dielectric surrounding media may induce undesirable interaction with bioanalytes for biosensing. Other approaches to exploit plasmonic abilities for enhanced chiroptical effects include the use of achiral nanostructures [64,65,66] to avoid noise signals. When excited with chiral incident fields, the corresponding plasmonic resonances exhibit enhanced chiral near-fields localized around the surface of these nanostructures. As in the case of localized surface plasmon resonances used in biosensing, these chiral near fields can have enhanced interactions with chiral bioanalytes attached to the nanoparticles. Limitations of this approach are mainly associated with the need for knowing the chiral handedness of the bioanalytes under study a priori.

In this review paper, we shall discuss the enhancement of chirality of the local, near-field, electromagnetic field using plasmonic nanostructures. For nanoparticles, CD signals are calculated using the corresponding extinction cross-sections, $Q_{\mathrm{ext}}$, defined as the sum of the absorption ($Q_{\mathrm{abs}}$) and scattering ($Q_{\mathrm{scat}}$) cross-sections, $Q_{\mathrm{ext}}=Q_{\mathrm{abs}}+Q_{\mathrm{scat}}$, for each circular polarization [35]

(5) $$\mathrm{CD}=\tan^{-1}\left(\frac{Q_{\mathrm{RCP}}-Q_{\mathrm{LCP}}}{Q_{\mathrm{RCP}}+Q_{\mathrm{LCP}}}\right).$$

For two-dimensional (2D) periodic arrangements of chiral plasmonic scatterers, named chiral plasmonic metasurfaces, CD signals are obtained using the transmittance/reflectance (*T*/*R*) of light going/coming through/from the structure for each polarization [35]

(6) $$\mathrm{CD}=\tan^{-1}\left(\frac{T_{\mathrm{RCP}}-T_{\mathrm{LCP}}}{T_{\mathrm{RCP}}+T_{\mathrm{LCP}}}\right),$$

where $T_{\mathrm{RCP}}$ ($R_{\mathrm{RCP}}$) and $T_{\mathrm{LCP}}$ ($R_{\mathrm{LCP}}$) are the transmitted (reflected) power intensities for RCP and LCP incident light.

## 3. Chiral Plasmonic Nanoparticles

Plasmonic chiral nanoparticles may be used to control circular polarization states of light, which is of practical interest for chemical and biological research owing to the strong optical interaction of molecules/biomolecules near plasmonic surfaces. The most obvious chiral nanoparticle one can imagine is the 3D nanohelix, described by a helical pitch and a coil diameter. Nanohelices can be grown from a surface stimulated by light to evolve according to the light’s handedness. The CD signal can be controlled through the number of helical loops, pitch size and coil diameter, as demonstrated theoretically and experimentally [19]. Significantly, chiral near- and far-fields can be produced in these structures even when excited with linearly polarized light (achiral) [19,20]. Various stimulation processes can be employed to produce these nanohelices [15], including Focused Ion Beam Induced Deposition (FIBID) with which the vertical pitch (the maximum diameter of the nanohelix) starts to increase above 20 keV. Below this threshold, the metallic ring does not grow, leaving Au rings on the surface [15,67]. Focused Electron Beam Induced Deposition (FEBID) has also been used to fabricate nanohelices, where the vertical pitch (VP) decreases at high electron energies because the continuous nucleation (process that assembly the structure) ceases, making the evolution impossible. Using low energies CD signals are limited due to the appearance of secondary structures, because of the high density of the electrons and superposition of the helix loops [15,67,68]. Another important limitation for these plasmonic nanoparticles reaching measurable CD signals is the need for precisely controlled pressure and temperature during growth. For example, the CD signal decreases from 0.3 to negligible values (≈0) when the temperature for nanoparticle growth increased from 170 K to 300 K [15]. These difficulties have stimulated research on new chiral plasmonic nanoparticles and nanostructures, some of which will be discussed next.

**Figure 4:**
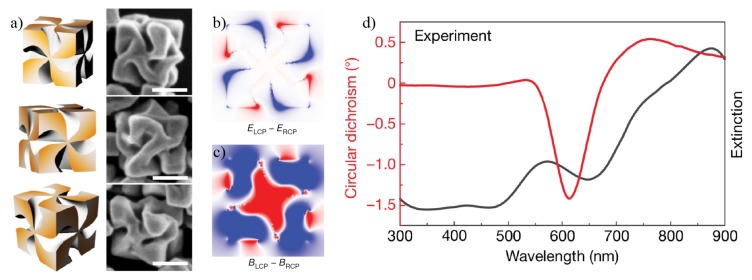
(**a**) Different three-dimensional perspectives (left) and SEM images (right) of a chiral plasmonic nanoparticle evolved from an octahedral seed. (**b**) Differential electric (*E*) and (**c**) magnetic *B* near-fields after successive illumination with LCP and RCP incident light. (**d**) Experimental results for CD (red) and extinction (black) spectra. Reproduced with permission from ref. [69]. Copyright 2018 Springer Nature.

One recent proposal consists of using *L*- or *D*-cysteine (*L*-Cys or *D*-Cys) to transfer their chirality to Au cube-shaped nanoparticles [69]. In this mechanism, *L*-Cys or *D*-Cys is attached to the cube’s faces, which in turn acquire an helicoidal shape inverse to the corresponding cysteine’s handedness, as shown in Figure 4a. *L* and *D* handedness refer to the Latin words *laevus* (left) and *dexter* (right), which are commonly used in chemical notation. Figure 4b,c show the differential electric ($E_{\mathrm{LCP}}-E_{\mathrm{RCP}}$) and magnetic ($B_{\mathrm{LCP}}-B_{\mathrm{RCP}}$) fields under sequentially applied LCP and RCP incident light. Figure 4c displays the corresponding experimental results for the CD signal in red, and the extinction (absorption) coefficient in black [69]. The CD signals using this approach are considerably higher than in previous proposals, but the peaks are wide, leading to a poor resolution.

**Figure 5:**
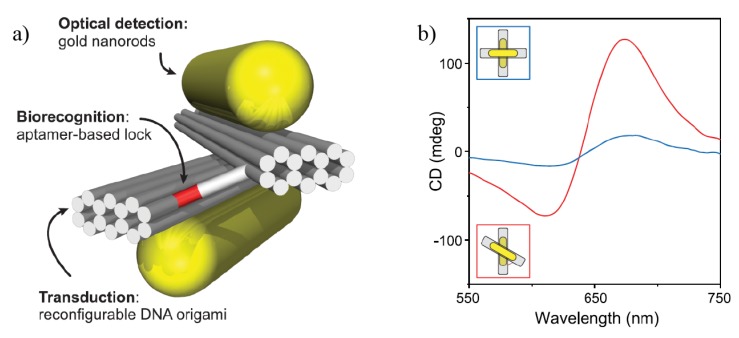
(**a**) A DNA-origami system with a bilayer composed of Au nanorods attached to aptamers (biorecognition elements). (**b**) CD spectra in orthogonal (blue line) and non-orthogonal (red line) configurations are presented. Reproduced with permission from ref. [72]. Copyright 2018 American Chemical Society.

CD can also be enhanced by combining the high affinity and selectivity of aptamers with largely enhanced electromagnetic near-fields in plasmonic nanoparticles [70,71,72]. The spatially reconfigurable DNA-origami nanofabrication technique is used to provide these aptamer-plasmonic assemblies with a chiroptical response. An example is given in Figure 5a, where a long synthetic DNA strand is used to perform molecular self-folding to crosslink spatially distant segments of a scaffold together. It is analogous to the art of folding and sculpting a flat sheet of paper, referred to as origami. Spatially distributed plasmonic nanoparticles on these building segments are used to control the near- and far-field radiation properties of the plasmonic arrangement. CD signals are produced when the nanoparticles follow a chiral geometry. Figure 5b shows that the CD signal can be tailored by manipulation of the relative orientation of nearby plasmonic nanorods, as depicted in the insets, making this proposal suitable for a wide range of analytes [71,72]. These origami-like structures are versatile in controlling CD peaks, but the intensity of the CD signal is small, thus making it difficult to apply to low analyte concentrations.

**Figure 6:**
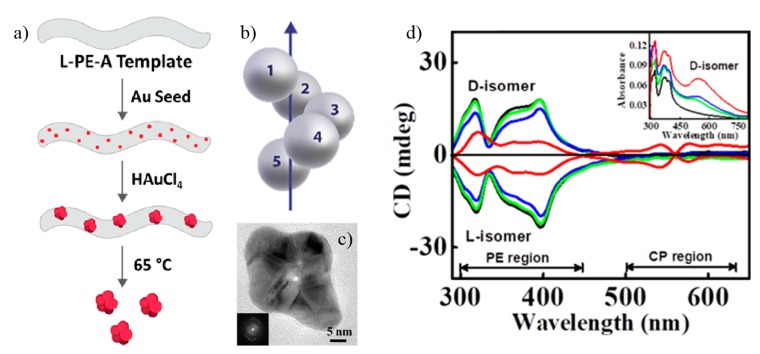
(**a**) Different steps for growing chiral nanoparticle arrangements along a polymer nanotube (used as a seed). (**b**) and (**c**) schematic illustration and TEM image of the resulting chiral Au nanostructure, respectively. (**d**) CD spectra for D- and L-isomer structures. The absorbance spectra are also shown in the inset. Reproduced with permission from ref. [78]. Copyright 2019 American Chemical Society.

Other strategies to control CD signals with only a few parameters or materials include self-assembly techniques and 3D polymer multilayers [71,72,73,74,75,76,77]. Gold nanoparticles deposited in a rotation sequence following a helical structure, analogue to the DNA, can be developed with nanotubes of phenyleneethynylene functionalized by *D*- or *L*-alanine (*D*- or *L*-PE-A) as depicted in Figure 6a [78]. Metallic nanospheres (seeds) are self-assembled on the nanotube’s surface in a helix-like structure with a rotation sequence after a proper thermal and chemical treatment. Structures like the one in Figure 6b are produced, displayed in the TEM image in Figure 6c. The corresponding CD signals are shown in Figure 6d, with their absorption spectra in the inset for the *D*-isomer case. An increased number of narrow peaks can be obtained with these systems, but their amplitudes are small and therefore sensing is only possible for large amounts of chiral samples. An analogous structure was already used for detection of amyloid fibrils in Parkinson’s disease down to nanomolar concentrations [79].

**Figure 7:**
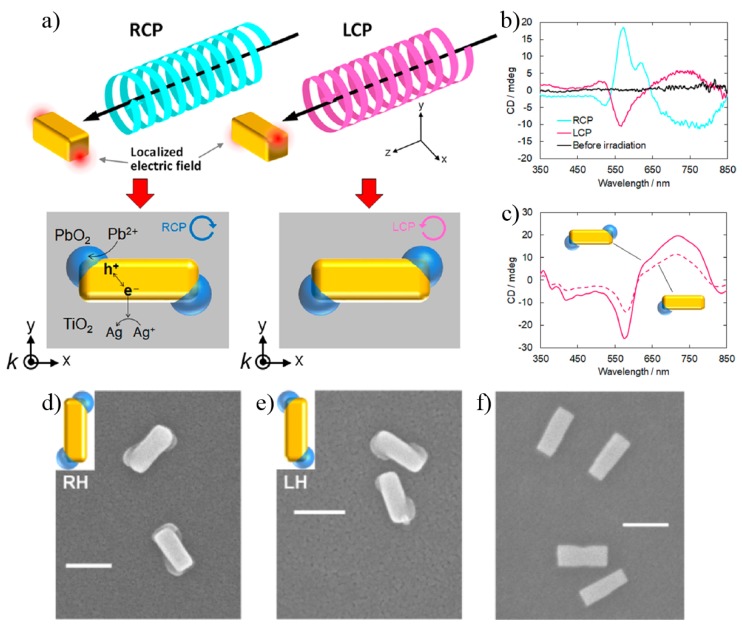
(**a**) Illustration of the deposition of a dielectric material (PbO${}_{2}$) via Pb${}^{2+}$ oxidation under CPL stimulus. (**b**) Substrate CD before the dielectric material deposition (black line) and after deposition under RCP (blue line) and LCP (red line) lights. (**c**) Comparison of CD spectra of a left-handed nanocuboid with one and two PbO${}_{2}$. SEM images of a right-handed (**d**), a left-handed (**e**) and before deposition (**f**) nanocuboids. Reproduced with permission from ref. [80]. Copyright 2018 American Chemical Society.

The chirality of circularly polarized light can be used as a sole chiral source to develop chiral plasmonic nanoparticles [80], as illustrated in the top section of Figure 7a. In this case, plasmon-induced charges separation (PICS) was employed in gold nanocuboids, used as precursors on a semiconducting TiO${}_{2}$ dielectric. CPL irradiation induced localized electric fields at specific corners of the cuboids immersed in a solution of Pb(NO${}_{3}$)${}_{2}$ and AgNO${}_{3}$. The chiral deposition of PbO${}_{2}$ on the corners of the cuboids is possible by the oxidation of Pb${}^{+2}$ based on PICS under LCP or RCP irradiation. The electric field hotspots on the nanocuboids depend on the incident light handedness. The chiroptical activity of these nanostructures was confirmed by CD measurements in Figure 7b, where the CD spectra are shown before deposition of the moieties (black line) and after irradiation with RCP (blue line) and LCP (red line). The results for numerical simulations are also shown in Figure 7c for a left-handed nanocuboid with one or two PbO${}_{2}$ moieties, respectively, as indicated by the insets. The SEM images of right-handed (RH), left-handed (LH) and without PbO${}_{2}$ deposition nanocuboids are shown in Figure 7d–f, respectively.

The examples discussed above demonstrate that relatively simple plasmonic nanostructures may offer a way for enantioselective absorption of light. However, they are limited owing to the low CD signals or wide peaks [78]. This has motivated a variety of new studies with top-down and bottom-up strategies to grow nanostructures [81], and fabrication of more complex structures such as chiral plasmonic gammadions, nanospirals, plasmonic oligomers, stereometamaterials, nano-barcodes, nano-ziguezags and nanohooks [14,82]. Since the optimal development of chiral plasmonic structures is still an open field, materials design based on computer simulations may become prominent in the next few years. For instance, one may consider the design of hybrid dielectric-plasmonic platforms to diminish the level of losses. Other approaches may include chiral resonant cavities, where extremely localized chiral near-fields are useful for enantioselective separation and probing of chiral molecules [83].

## 4. Chiral Plasmonic Metasurfaces

Several challenges must be faced to employ plasmonic chiral nanoparticles in biosensing platforms, as discussed above. An alternative to these nanoparticles is to use chiral plasmonic metamaterials that exhibit intriguing optical phenomena, not found in natural materials, such as optical rotatory dispersion (ORD) and strong circular dichroism (CD). In these systems, the size and separation of building elements must be smaller than the working wavelength to have an effective medium. In the scenario where the metamaterial is made as a film, sufficiently thin to be comparable with the incident wavelength, it can be considered to be a metasurface, i.e., a planar metamaterial that not only allows for the same unusual optical phenomena but may also display strong surface plasmon resonance (SPR) with collective electronic oscillations [84,85]. Taichi-like chiral plasmonic metasurfaces for preferential absorption of RCP or LCP light were developed to work in the infrared region [86,87], where the polarization selectivity of these structures was demonstrated by switching the image contrast of the Taichi logo with changes in the polarization of the incident light (in the resonance wavelength). Merging these plasmonic and chiral features with surface functionalization techniques may open new routes for chiral plasmonic biosensing in the reflection mode.

**Figure 8:**
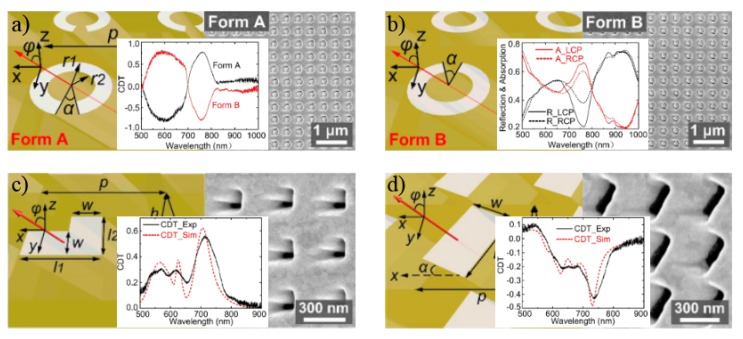
(**a**) Form A of the slanted split-ring aperture (left) with the SEM image (right) and the CDT (circular dichroism in transmission) spectra for both forms (center). (**b**) Form B of the slanted split-ring aperture (left), the SEM image (right) and the reflection and absorption spectra for Form A under LCP and RCP incidence light (center). (**c**) A L-shaped aperture metasurface (left), the SEM image (right) and the CDT from experiments and simulation (center). (**d**) A rectangular aperture metasurface (left), SEM image (right) and the CDT spectra from experiments and simulations (center). Reproduced with permission from ref. [89]. Copyright 2017 American Chemical Society.

A relatively simple technique to develop plasmonic metasurfaces is the focused ion beam (FIB) milling process, which uses highly focused ion beams such as Ga${}^{+}$ to locally sputter or mill a sample surface inside a vacuum chamber [88]. Figure 8 illustrates four chiral plasmonic metasurfaces made by FIB, with the corresponding SEM images and CD signals shown in the insets. Figure 8a,b show the two opposite handedness mirror images (with respect to the xz-plane), named forms A and B, of a slanted split-ring aperture [89]. These structures are easily fabricated by simply tilting the sample substrate relative to the ion beam, as depicted by the angle φ between the sample surface normal and the incident direction of the ion beam in Figure 8. Hence, highly uniform slanted nanoapertures with arbitrary shapes can be molded [89,90,91], as shown in Figure 8c,d for slanted L-shape and rectangular nanoapertures, respectively. Transmission along these structures is determined by the strong plasmonic enantioselective chiral near-field flow along the apertures, thus favoring the passage of one of the CPL handedness while reflecting the other one. This phenomenon can be tailored and tuned by varying the tilting angle (φ), thickness (*h*) of the surface and angle α (for split-ring apertures). Then, it may be exploited for biosensing after proper functionalization of the metallic surface, where transmission through the apertures will suffer changes due to the handedness of the adsorbed molecules [89]. Please note that the CD signal in transmission is measured as the differential transmission after successively applied RCP and LCP incident light [89,92], i.e., $\mathrm{CDT}=\Delta T=T_{\mathrm{RCP}}-T_{\mathrm{LCP}}$.

**Figure 9:**
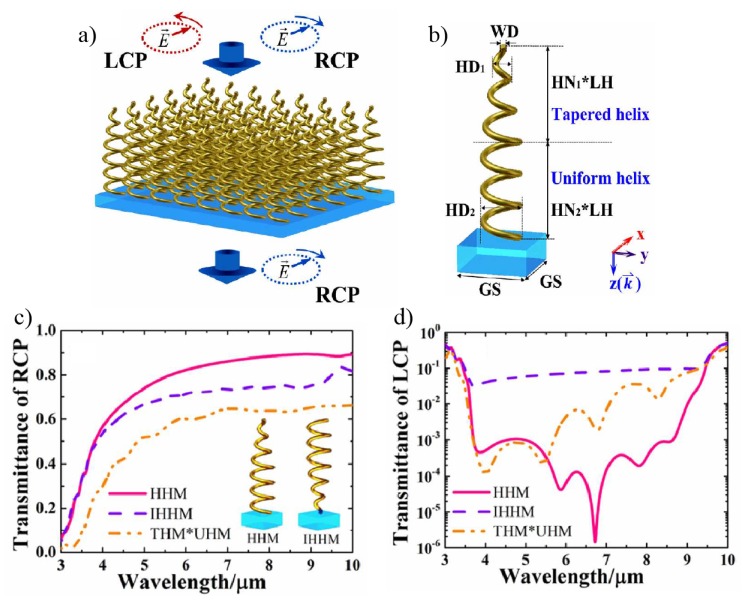
(**a**). Illustration of hybrid nanohelices array on a substrate and the polarization filter potential. (**b**) A single hybrid nanohelix, made by a uniform (UHM) and a tapered (THM) helices, with their own diameter (HD${}_{1}$ and HD${}_{2}$). Transmittance spectra of RCP (**c**) and LCP (**d**) of hybrid (HHM), inverted hybrid (IHHM) and the junction of THM and UHM helices. Reproduced with permission from ref. [93]. Copyright 2016 American Chemical Society.

CD signal enhancement may also be achieved with arrays of hybrid nanohelices [93], chiral plasmonic metasurfaces containing semiconductor colloidal quantum dots (CQDs) [94], N- and V-shaped [92] and multilayers [59]. In the first case, arrays of hybrid helix metamaterials (HHM) are fabricated by coupling uniform (UHM) and tapered (THM) helix metamaterials [93]. This is illustrated in Figure 9a where the nanohelices are left-handed. Figure 9b shows the single cell and its composition, UHM in the bottom and THM in the top, with the same helix wire diameter (WD), length-period (LH) and grid spacing (GS) for both, but different helix diameters (from HD${}_{1}$ to HD${}_{2}$ for THM and HD${}_{2}$ for UHM). The transmittance spectra of HHM, inverted HHM (IHHM) and combination of THM and UHM are shown in Figure 9c,d, for RCP and LCP, respectively. As the optical response for RCP is higher than for LCP (decreases almost to micro scale), this metamaterial has a potential use for polarization filter, represented in Figure 9a, where the left-handed light is filtered.

**Figure 10:**
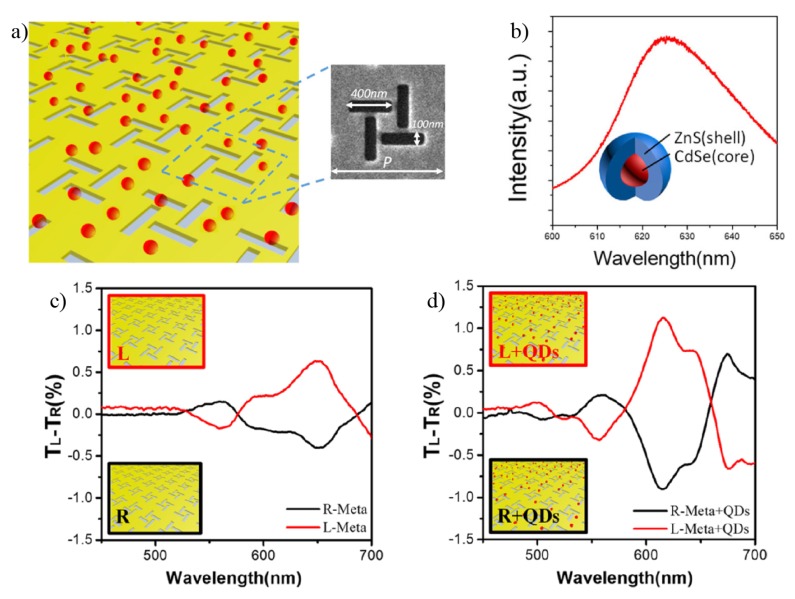
(**a**) Illustrative representation of a hybrid CQDs-plasmonic-metasurface. The inset shows a SEM image of the plasmonic unit cell. (**b**) Photoluminescence spectrum of core-shell CQDs, schematically shown in the inset. For comparative purposes, (**c**) and (**d**) show the results for CDT spectra without and with CQDs. Reproduced with permission from ref. [94]. Copyright 2019 John Wiley and Sons.

In Figure 10, chiral plasmonic metasurfaces are used for chiral functionalization of achiral CdSe/ZnS core-shell quantum dots. The metasurface consists of a two-dimensional periodic arrangement of perpendicular nanoslits, as illustrated in Figure 10a. It was fabricated by FIB milling process on a 100 nm thick gold film, with the corresponding unit cell shown in the SEM image in the inset of Figure 10a. In this approach, the absorption and emission processes in CQDs are resonantly coupled with the localized surface plasmon resonance - for enhanced light-matter interaction - in the chiral plasmonic metasurface for CD signal enhancement. The resonance matching between the CQDs and the chiral metasurface is tuned by altering the lattice period of the superlattice. In Figure 10b is shown the photoluminescence spectrum of CdSe/ZnS QDs on a glass substrate with a peak at 623 nm. The corresponding core/shell QD structure is schematically illustrated in the inset. Figure 10c,d show a comparative analysis of the $\mathrm{CDT}=T_{\mathrm{RCP}}-T_{\mathrm{LCP}}$ signal with and without CQDs, respectively, for both opposite handedness mirror images of the metasurface (named forms R and L), where a clear CD signal enhancement can be seen [94].

**Figure 11:**
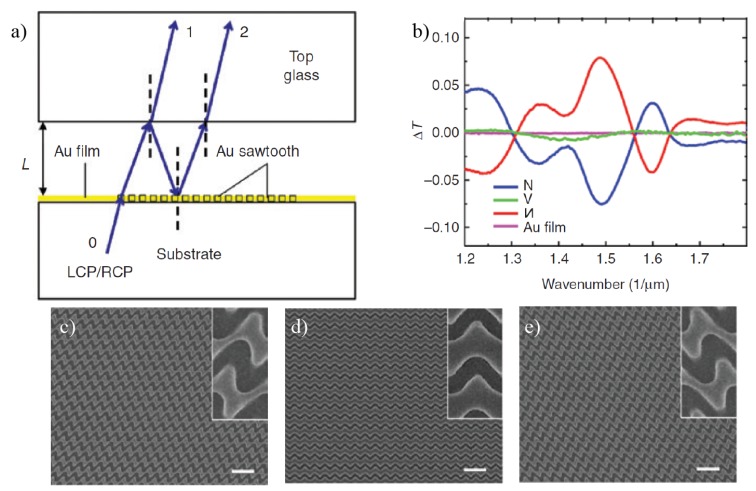
(**a**) Pictorial view of an Au-sawtooth metasurface surrounded by glass for circular phase-dichroism measurements. (**b**) CDT spectra from N-shaped, И-shaped, V-shaped and Au film surfaces. (**c**)–(**e**) The corresponding SEM images of the Au-metasurfaces. Reproduced with permission from ref. [92]. Copyright 2019 De Gruyter.

Figure 11a shows the schematics of the experimental setup for measuring the transmission and reflection phase of CPL in a Fabry-Perot resonant cavity. Figure 11b shows the transmission difference CDT through N-shaped, И-shaped, V-shaped and Au film metasurfaces, with N-shaped chiral plasmonic surfaces [92] exhibiting CDT of the order of 10% (higher than the resonantly coupled CQDs). The measurements for N-shaped and И-shaped plasmonic surfaces demonstrated the chiral nature of these geometries. The corresponding SEM images are shown in Figure 11c–e [92].

**Figure 12:**
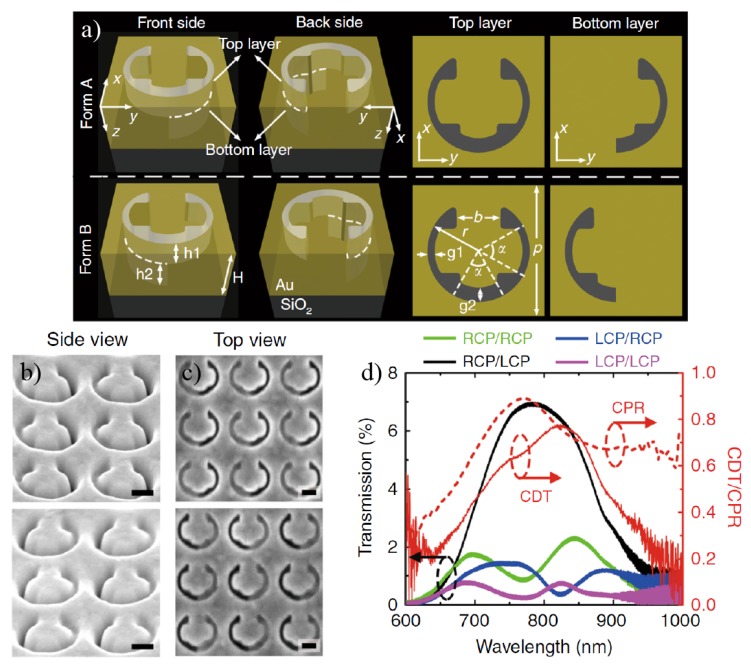
(**a**) Enantiomeric multilayer nanoapertures (forms A and B) are depicted. The total Au film thickness is H=180 nm. Top and bottom building layers have aperture thicknesses of h1=80 nm and h2=100 nm. Other geometrical parameters are taken as p=360 nm, r=140 nm, b=120 nm, $\alpha =60^{\circ }$, g1=20 nm, and g2=40 nm. Light is considered to be vertically impinging onto the top layer and transmitted out of the bottom layer. (**b**) and (**c**) are the sideview ($52^{\circ }$) and top view SEM images of the multilayer nanoapertures fabricated using the FIB milling method. The scale bar is 100 nm. (**d**) Experimental values of the transmission, CDT and CPR spectra of the nanoapertures in Form A for different incident/output handedness combinations. Reproduced with permission from ref. [59]. Copyright 2018 Springer Nature.

The example of a multilayer surface [59] is illustrated in Figure 12a with an aperture differing from the split ring already mentioned, but also fabricated with a focused ion beam milling process. In this case, the handedness of the structure is ruled by the bottom layer, where only half-side of the structure continues in the second layer. The SEM images with the side and top view of these structures are shown in Figure 12b,c, respectively. The result is a multilayer structure with the ability to transmit and convert the polarization of the incident light. Figure 12d shows the ratios for the possible combinations of incident/transmitted light (LCP/LCP, RCP/RCP, LCP/RCP and RCP/LCP), on a structure following Form A, which exhibits a high CDT and cross polarization ratio (CPR). The maximum transmission efficiency is found for the converted polarization, i.e., transmitted light has mainly the opposite polarization to the incident one.

Despite very high CD signals from the systems in Figure 8, their associated peaks are broad, thus hampering applications for detection of small analytes or at low concentration levels. On the other hand, narrower peaks in Figure 10 and Figure 11 make these systems promising for biosensing applications. However, their CD signals are very small. Therefore, more research is needed to find proper systems for this kind of application. Although other approaches, including the use of three-dimensional metamaterials [95], *b*-type bilayer [96] and monolayer [96] metasurfaces and hybrid plasmonic-dielectric metasurfaces [62,97], exhibited a strongly localized electromagnetic near-field, highly desirable for biosensing, they were mainly set for polarization selective near-perfect absorption and quasi-null reflecting devices [98,99].

## 5. Summary and Outlook

**Figure 13:**
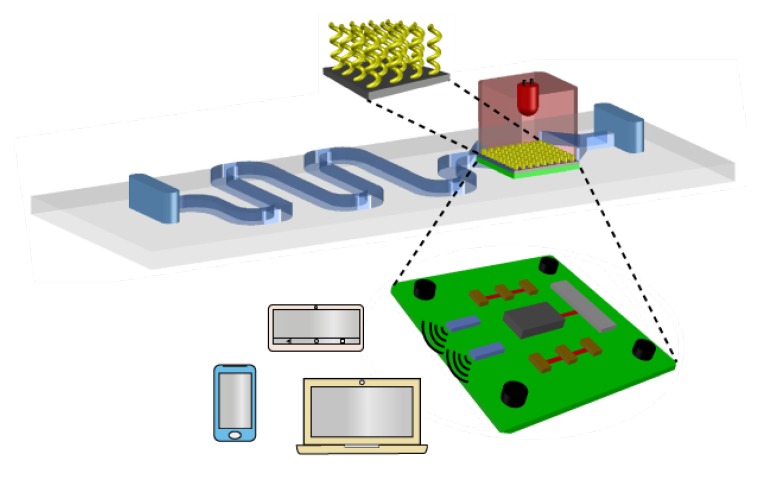
Chiral plasmonics for PoC devices integrated with IoT: Biosensing device working as a PoC device that collects samples, e.g., blood, treats them in the microfluidic system (blue way) to reach the plasmonic chamber. The chiral plasmonic structure with suitable functionalization will capture the enantiomer of interest. The optical response following the light incidence will be transduced into electrical pulses by the transductor (grey component in the circuit board), amplified in a three-stage amplification (brown components) and emitted to near or remote device as a radiofrequency (RF) signal (the antennas are the blue components).

The use of concepts and strategies of chiral plasmonics has been shown promising, despite the limitations discussed in this review paper. Indeed, the recent advances in nanofabrication, numerical modeling and characterization tools, allow us to envisage the merge of chiral plasmonics and microfluidic systems for the development of rapid and precise lab-on-a-chip biosensors, commonly referred to as point-of-care (PoC) devices [100,101,102,103,104,105,106]. Microfluidics alone has also been applied for characterization of biological structures [107], while plasmonic nanostructures may be used in PoC systems to detect ions, antigens, acids and other small molecules [108,109,110,111,112,113]. Brolo et al. discussed the next-generation of biosensing, emphasizing the need for remote data transfer and how plasmonics will be integrated with PoC technology to develop itself in new areas [114]. In Figure 13 we illustrate a potential next-generation model of a plasmonic biosensing device integrated with an Internet-of-Things (IoT) system, resulting in a PoC device that can analyze a fluid, e.g., blood, saliva, sweat or urine. For chiral sensing, the analyte will be carried through the microfluidic device and be treated, as illustrated by the route in blue (this is only one of the possibilities). Functionalization of nanoparticles surfaces will be needed for improved specificity. The waste will be discarded at the end of the microfluidic system. A proper washing mechanism must be also included in these platforms to allow for reuse to avoid contamination and reduce costs. After proper light excitation, signal processing should take place on an integrated circuit board, shown in green color. This board must also contain a transducer, illustrated in grey color, to translate the optical to electric response, which may in principle need to be amplified (brown component, a three-stage amplifier). Then, the signal will be transmitted as converted to radiofrequency (RF) signal by the antennas (illustrated by blue rectangular components), allowing a remote or local communication between the biosensor and the electronics that will receive the RF signal for real-time data processing and monitoring [115,116,117]. When huge amounts of data are collected in e-Health servers through IoT integration [118], artificial intelligence (AI) and big-data technologies [119,120] are expected to help in the development of faster, efficient, and improved systems that may apply to diseases such as Parkinson’s and Alzheimer’s diseases [121,122].

The generic platform in Figure 13 may be applied beyond chiral plasmonics, being a possible ingredient in a new healthcare paradigm where diagnostics and treatment are expected to be individualized, with continuous monitoring of patients’ health [123,124,125,126,127,128,129,130]. In this approach, an active participation of the patient for disease management is expected via wearable and/or handheld devices, for self-assessment through information sharing with peer groups and specialized medical centers, thus reducing costs of regional and global healthcare systems [121,122]. Recent proposals in this direction have been used in pharmacology to probe patient’s reaction after administering medicines [131]. One of the major objectives in PoC devices is their integration with telecommunications for remote analysis and diagnostics, where the Internet-of-Things (IoT), i.e., the connection of any physical object to the Internet, and big-data analysis are envisaged for future diagnosis and treatments [132,133,134,135]. The associated risks of this technology and regulations needed for an efficient, fair working paradigm are also being considered [100,101,102].

## References

[1] McConathy J., Owens M.J. (2013). Stereochemistry in Drug Action. Primary Care Companion J. Clin. Psychiatry.

[2] Singh K., Shakya P., Kumar A., Alok S., Kamal M., Singh S.P. (2014). Stereochemistry and its role in drug design. Int. J. Pharm. Sci. Res..

[3] Singh R., Nalwa H.S. (2011). Medical Applications of Nanoparticles in Biological Imaging, Cell Labeling, Antimicrobial Agents and Anticancer Nanodrugs. J. Biomed. Nanotechnol..

[4] Patching S.G. (2014). Surface plasmon resonance spectroscopy for characterization of membrane protein-ligand interactions and its potential for drug discovery. Biochim. Biophys. Acta.

[5] Du X., Zhou J., Wang J., Zhou R., Xu B. (2016). Chirality Controls Reaction-Diffusion of Nanoparticles for Inhibiting Cancer Cells. Chem. Nano Mat..

[6] Jazayeria M.H., Aghaieb T., Avanc A., Vatankhahd A., Ghaffarid M.R.S. (2018). Colorimetric detection based on gold nano particles (GNPs): An easy, fast, inexpensive, low-cost and short time method in detection of analytes (protein, DNA, and ion). Sens. Bio-Sens. Res..

[7] Nguyen L.A., He H., Pham-Hyu C. (2006). Chiral Drugs: An Overview. Int. J. Biomed. Sci..

[8] Smith S.W. (2009). Chiral Toxicology: It’s the Same Thing...Only Different. Toxicol. Sci..

[9] Tokunaga E., Yamamoto T., Ito E., Shibata N. (2018). Understanding the Thalidomide Chirality in Biological Processes by the Self-disproportionation of Enantiomers. Sci. Rep..

[10] Dobson C.M. (2003). Protein Folding and Misfolding. Nature.

[11] Kelly S.M., Jess T.J., Price N.C. (2005). How to Study Proteins by Circular Dichroism. Biochim. Biophys. Acta.

[12] Lieberman I., Shemer G., Fried T., Kosower E.M., Markovich G. (2008). Plasmon-Resonance-Enhanced Absorption and Circular Dichroism. Angew. Chem. Int. Ed..

[13] Hendry E., Carpy T., Johnston J., Popland M., Mikhaylovskiy R.V., Lapthorn A.J., Kelly S.M., Barron L.D., Gadegaard N., Kadodwala M. (2010). Ultrasensitive detection and characterization of biomolecules using superchiral fields. Nat. Nanotechnol..

[14] Mark A.G., Gibbs J.G., Lee T., Fischer P. (2013). Hybrid nanocolloids with programmed three-dimensional shape and material composition. Nat. Mater..

[15] Esposito M., Tasco V., Cucuna M., Todisco F., Benedetti A., Tarantini I., Giorgi M., Sanvitto D., Passaseo A. (2014). Nanoscale 3D Chiral Plasmonic Helices with Circular Dichroism at Visible Frequencies. ACS Photonics.

[16] Nair G., Singh H.J., Paria D., Venkatapathi M., Ghosh A. (2014). Plasmonic Interactions at Close Proximity in Chiral Geometries: Route toward Broadband Chiroptical Response and Giant Enantiomeric Sensitivity. J. Phys. Chem. C.

[17] Gutsche P., Mäusle R., Burger S. (2016). Locally Enhanced and Tunable Optical Chirality in Helical Metamaterials. Photonics.

[18] Zu S., Bao Y., Fang Z. (2016). Planar plasmonic chiral nanostructures. Nanoscale.

[19] Schäferling M., Yin X., Engheta N., Giessen H. (2014). Helical Plasmonic Nanostructures as Prototypical Chiral Near-Field Sources. ACS Photonics.

[20] Schäferling M., Engheta N., Giessen H., Weiss T. (2016). Reducing the Complexity: Enantioselective Chiral Near-Fields by Diagonal Slit and Mirror Configuration. ACS Photonics.

[21] Hu L., Huang Y., Pan L., Fang Y. (2017). Analyzing Intrinsic Plasmonic Chirality by Tracking the Interplay of Electric and Magnetic Dipole Modes. Sci. Rep..

[22] Mejía-Salazar J.R., Oliveira O.N. (2018). Plasmonic Biosensing. Chem. Rev..

[23] Bochenkov V.E., Shabatina T.I. (2018). Chiral Plasmonic Biosensors. Biosensors.

[24] Ma W., Xu L., Wang L., Xu C., Kuang H. (2019). Chirality-Based Biosensors. Adv. Funct. Mater..

[25] Pal S., Deng Z., Ding B., Yan H., Liu Y. (2010). DNA-Origami-Direct Self-Assembly of Discrete Silver-Nanoparticle Architectures. Angew. Chem. Int..

[26] Frank B., Yin X., Schäferling M., Zhao J., Hein S.M., Braun P.V., Giessen H. (2013). Large-Area 3D Chiral Plasmonic Structures. ACS Nano.

[27] Kühler P., Roller E.M., Schreiber R., Liedl T., Lohmüller F.J. (2014). Plasmonic DNA-Origami Nanonantennas for Surface-Enhanced Raman Spectroscopy. Nano Lett..

[28] Song J.C.W., Rudner M.S. (2016). Chiral plasmons without magnetic field. Proc. Natl. Acad. Sci. USA.

[29] Wang Z., Jia H., Yao K., Cai W., Chen H., Liu Y. (2016). Circular Dichroism Metamirrors with Near-Perfect Extinction. ACS Photonics.

[30] Schnell M., Sarriugarte P., Neuman T., Khanikaev A.B., Shvets G., Aizpurua J., Hillenbrand R. (2016). Real-Space Mapping of the Chiral Near-Field Distributions in Spiral Antennas and Planar Metasurfaces. Nano Lett..

[31] Wang Y., Deng J., Wang G., Fu T., Qu Y., Zhang Z. (2016). Plasmonic chirality of L-shaped nanostructure composed of two slices with different thickness. Opt. Express.

[32] Li Z., Wang W., Rosenmann D., Czaplewski D.A., Yang X., Gao J. (2016). All-metal structural color printing based on aluminum plasmonic metasurfaces. Opt. Express.

[33] Luo Y., Chi C., Jiang M., Li R., Zu S., Li Y., Fang Z. (2017). Plasmonic Chiral Nanostructures: Chiroptical Effects and Applications. Adv. Opt. Mater..

[34] Zhao Y., Askarpour A.N., Sun L., Shi J., Li X., Alú A. (2017). Chirality detection of enantiomers using twisted optical metamaterials. Nat. Commun..

[35] Mohammadi E., Tsakmakidis K.L., Askarpour A.N., Dehkhoda P., Tavakoli A., Altug H. (2018). Nanophotonic Platforms for Enhanced Chiral Sensing. ACS Photonics.

[36] Woźniak P., De Leon I., Höflich K., Haverkamp C., Christiansen S., Leuchs G., Banzer P. (2018). Chiroptical response of a single plasmonic nanohelix. Opt. Express.

[37] Kong X.T., Khorashad L.K., Wnag Z. (2018). Photothermal Circular Dichroism Induced by Plasmon Resonances in Chiral Metamaterial Absorbers and Bolometers. Nano Lett..

[38] Kaniewska M., Trojanowicz M. (2011). Chiral Biosensors and Immunosensors. Biosensors: Emerging Materials and Applications.

[39] Murugkar S., Leon I.D., Horton M., Qassin H., Leach J., Boyd R.W. (2013). Planar chiral metamaterials for biosensing applications. Plasmonics Biol. Med. X.

[40] Wu T., Ren J., Wang R., Zhang X. (2014). Competition of Chiroptical Effect Caused by Nanostructures and Chiral Molecules. J. Phys. Chem. C.

[41] Kailasa S.K., Koduru J.R., Desai M.L., Park T.J., Singhal R.K., Basu H. (2018). Recent progress on surface chemistry of plasmonic metal nanoparticles for colorimetric assay of drugs in pharmaceutical and biological samples. TrAC Trends Anal. Chem..

[42] Xu L.L., Zhang H.F., Li M., Ng S.W., Feng J.H., Mao G.G. (2018). Chiroptical Activity from an Achiral Biological Meta-Organic Framework. J. Am. Chem. Soc..

[43] Tang Y., Cohen A.E. (2010). Optical Chirality and Its Interaction with Matter. Phys. Rev. Lett..

[44] Harris R.A. (1965). On the Optical Rotary Dispersion of Polymers. J. Chem. Phys..

[45] Lipkin D.M. (1964). Existence of a New Conservation Law in Electromagnetic Theory. J. Math. Phys..

[46] Poulikakos L.V., Thureja P., Stollmann A., de Leo E., Norris D.J. (2018). Chiral Light Design and Detection Inspired by Optical Antenna Theory. Nano Lett..

[47] Pham A., Zhao A., Genet C., Drezet A. (2018). Optical Chirality Density and Flux Measured in the Local Density of States of Spiral Plasmonic Structures. Phys. Rev. A.

[48] Aba T., Qu Y., Abudukelimu A., Ullah H., Zhang Z. (2019). Chiral Response of a Metasurface Composed of Nanoholes and Tilted Nanorods. Appl. Opt..

[49] Rajaei M., Zeng J., Albooyeh M., Kamandi M., Hanifeh M., Capolino F., Wickramasinghe H.K. (2019). Giant Circular Dichroism at Visible Frequencies Enabled by Plasmonic Ramp-Shaped Nanostructures. ACS Photonics.

[50] Gilroy C., Hashiyada S., Endo K., Karimullah A.S., Barron L.D., Okamoto H., Togawa Y., Kadodwala M. (2019). Roles of Superchirality and Interference in Chiral Plasmonic Biodetection. J. Phys. Chem. C.

[51] Chen Y., Gao J., Yang X. (2019). Chiral Grayscale Imaging with Plasmonic Metasurfaces of Stepped Nanoapertures. Adv. Opt. Mater..

[52] Li Z., Liu C., Rong X., Luo Y., Chen H., Zheng L., Lin F., Shen B., Gong Y., Zhang S. (2018). Tailoring MoS_2_ Valley-Polarized Photoluminescence with Super Chiral Near-Field. Adv. Mater..

[53] Tseng M.L., Lin Z.-H., Kuo H.Y., Huang T.-T., Huang Y.T., Chung T.L., Chu C.H., Huang J.-S., Tsai D.P. (2019). Stress-Induced 3D Chiral Fractal Metasurface for Enhanced and Stabilized Broadband Near-Field Optical Chirality. Adv. Opt. Mater..

[54] Champi H.A.A., Bustamante R.H., Salcedo W.J. (2019). Optical Enantioseparation of Chiral Molecules Using Asymmetric Plasmonic Nanoapertures. Opt. Mater. Exp..

[55] Wang Z., Wang Y., Adamo G., Teh B.H., Wu Q.Y.S., Teng J., Sun H. (2016). A Novel Chiral Metasurface with Controllable Circular Dichroism Induced by Coupling Localized and Propagating Modes. Adv. Opt. Mater..

[56] Qu Y., Huang L., Wang L., Zhang Z. (2017). Giant circular dichroism induced by tunable resonance in twisted Z-shaped nanostructure. Opt. Exp..

[57] Kelly C., Khorashad L.K., Gadegaard N., Barron L.D., Govorov A.O., Karimullah A.S., Kadodwala M. (2018). Controlling Metamaterial Transparency with Superchiral Fields. ACS Photonics.

[58] Tang Y., Huang Y., Qv L., Fang Y. (2018). Electromagnetic Energy Redistribution in Coupled Chiral Particle Chain-Film System. Nanoscale Res. Lett..

[59] Chen Y., Yang X., Gao J. (2018). Spin-Controlled Wavefront Shaping with Plasmonic Chiral Geometric Metasurfaces. Light Sci. Appl..

[60] Lu F., Zhang W., Zhang J., Zhang L., Xue T., Liu M., Meng C., Mao D., Mei T. (2019). Dynamic manipulation of optical chirality for gammadion nanostructures. Appl. Phys. Exp..

[61] Chen Z., Chen S., Wang Y., Xiao L. (2019). Tunable atom-trapping based on a plasmonic chiral metamaterial. Nanophotonics.

[62] Yin S., Ji W., Xiao D., Li Y., Li K., Yin Z., Jiang S., Shao L., Luo D., Liu Y.J. (2019). Intrinsically or extrinsically reconfigurable chirality in plasmonic chiral metasurfaces. Opt. Commun..

[63] Chen Y., Yang X., Gao J. (2019). 3D Janus Plasmonic Helical Nanoapertures for Polarization-Encrypted Data Storage. Light Sci. Appl..

[64] Chen L., Zheng J., Feng J., Qian Q., Zhou Y. (2019). Reversible modulation of plasmonic chiral signals of achiral gold nanorods using a chiral supramolecular template. Chem. Commun..

[65] Hashiyada S., Narushima T., Okamoto H. (2019). Active Control of Chiral Optical near Fields on a Single Metal Nanorod. ACS Photonics.

[66] Horrer A., Zhang Y., Gérard D., Béal J., Kociak M., Plain J., Bachelot R. (2020). Local Optical Chirality Induced by Near-Field Mode Interference in Achiral Plasmonic Metamolecules. Nano Lett..

[67] Reyntjens S., Puers R. (2000). Focused ion beam induced deposition: Fabrication of three-dimensional microstructures and Young’s modulus of the deposited material. J. Micromech. Microeng..

[68] Huth M., Porrati F., Dobrovolskiy O.V. (2018). Focused electron beam induced deposition meets materials science. Microelectron. Eng..

[69] Lee H.E., Ahn H.Y., Mun J., Lee Y.Y., Kim M., Cho N.H., Chang K. (2018). Amino-acid- and peptide-directed synthesis of chiral plasmonic gold nanoparticles. Nature.

[70] Schneider F., Möritz N., Dietz H. (2019). The sequence of events during folding of a DNA origami. Sci. Adv..

[71] Zhou C., Duan X., Liu N. (2015). A plasmonic nanorod that walks on DNA origami. Nat. Commun..

[72] Huang Y., Nguyen M., Natarajan A.K., Nguyen V.H. (2018). A DNA Origami-Based Chiral Plasmonic Sensing Device. Appl. Mater. Interfaces.

[73] Shemer G., Krichevski O., Markovich G., Molotsky T., Lubitz T., Kotyar A.B. (2006). Chirality of Silver Nanoparticles Synthesized on DNA. J. Am. Chem. Soc..

[74] Swasey S.M., Karimova M., Aikens C.M., Schultz D.E., Simon A.J., Gwinn E.G. (2014). Chiral Electronic Transitions in Fluorescent Silver Cluster Stabilized by DNA. ACS Nano.

[75] Schreiber R., Luong N., Fan Z., Kuzyk A., Nickels P.C., Zhang T., Smith D.M., Yurke B., Kuang W., Govorov A.O. (2013). Chiral plasmonic DNA nanostructures with switchable circular dichroism. Nat. Commun..

[76] Fan Z., Govorov A.O. (2011). Helical Metal Nanoparticle Assemblies with Defects: Plasmonic Chirality and Circular Dichroism. J. Phys. Chem..

[77] Kuzyk A., Schreiber R., Fan Z., Pardatscher G., Roller E.M., Högele A., Simmel F.C., Govorov A.O., Liedl T. (2012). DNA-based self-assembly of chiral plasmonic nanostructures with tailored optical response. Nature.

[78] George J., Kar S., Anupriya E.S., Somasundaran S.M., Das A.D., Sissa C., Painelli A., Thomas K.G. (2019). Chiral Plasmons: Au Nanoparticle Assemblies on Thermoresponsive Organic Template. ACS Nano.

[79] Kumar J., Eraña H., López-Martínez E., Claes N., Martín V.F., Solís D.M., Bals S., Cortajarena A.L., Castilla J., Liz-Marzán L.M. (2018). Detection of Amyloid Fibrils in Parkinson’s Disease Using Plasmonic Chirality. Proc. Natl. Acad. Sci. USA.

[80] Saito K., Tatsuma T. (2018). Chiral Plasmonic Nanostructures Fabricated by Circularly Polarized Light. Nano Lett..

[81] Hentschel M., Schäferling M., Duan X., Giessen H., Liu N. (2017). Chiral plasmonics. Sci. Adv..

[82] Schäferling M., Dregely D., Hentschel M., Giessen H. (2012). Tailoring Enhanced Optical Chirality: Design Principles for Chiral Plasmonic Nanostructure. Phys. Rev. X.

[83] Zhao Y., Saleh A.A.E., Dionne J.A. (2016). Enantioselective Optical Trapping of Chiral Nanoparticles with Plasmonic Tweezers. ACS Photonics.

[84] Chen H.T., Taylor A.J., Yu N. (2016). A review of metasurfaces: Physics and applications. Rep. Prog. Phys..

[85] Glybovski S.B., Tretyakov S.A., Belov P.A., Kivshar Y.S., Simovski C.R. (2016). Metasurfaces: From Microwaves to Visible. Phys. Rep..

[86] Ouyang L., Wang W., Rosenmann D., Czaplewski D.A., Gao J., Yang X. (2018). Near-infrared chiral plasmonic metasurface absorbers. Opt. Express.

[87] Huang Y., Yao Z., Wang Q., Hu F., Xu X. (2015). Coupling Tai Chi Chiral Metamaterials with Strong Optical Activity in Terahertz Region. Plasmonics.

[88] Sezen M. (2016). Focused Ion Beam (FIB)—Novel Methodologies and Recent Applications for Multidisciplinary Sciences. Mod. Electron Microsc. Phys. Life Sci..

[89] Chen Y., Gao J., Yang X. (2018). Chiral Metamaterials of Plasmonic Slanted Nanoapertures with Symmetry Breaking. Nano Lett..

[90] Ndao A., Belkhir A., Salut R., Baida F.I. (2013). Slanted annular aperture arrays as enhanced-transmission metamaterials: Excitation of the plasmonic transverse electromagnetic guided mode. Appl. Phys. Lett..

[91] Lv J., Khoo E.H., Leong E.S.P., Hu L., Jiang X., Li Y., Luo D., Si G., Liu Y.J. (2017). Maskless fabrication of slanted annular aperture arrays. Nanotechnology.

[92] Zhang R., Zhao Q., Wang X., Gao W., Li J., Tam W.Y. (2019). Measuring circular phase-dichroism of chiral metasurface. Nanophotonics.

[93] Ji R., Wang S.-W., Liu X., Guo H., Lu W. (2016). Hybrid helical metamaterials for giant and ultra-wide circular dichroism. ACS Photonics.

[94] Wang Z., Wnag Y., Adamo G., Teng J., Sun H. (2019). Induced Optical Chirality and Circularly Polarized Emission from Achiral CdSe/ZnS Quantum Dots via Resonantly Coupling with Plasmonic Chiral Metasurfaces. Laser Photonics Rev..

[95] Fang Y., Verre R., Shao L., Nordlander P., Käll M. (2016). Hot Electron Generation and Cathodoluminescence Nanoscopy of Chiral Split Ring Resonators. Nano Lett..

[96] Liu H., Shang Z., Wu X., Dou C., Zhang J. (2019). Tunable circular dichroism of bilayer b-type chiral nanostructure. Opt. Commun..

[97] Rifat A.A., Rahmani M., Xu L., Miroshnichenko E. (2018). Hybrid Metasurface Based Tunable Near-Perfect Absorber and Plasmonic Sensor. Materials.

[98] Zhang S., Wei H., Bao K., Håkanson U., Halas N.J., Nordlander P., Xu H. (2011). Chiral Surface Plasmon Polaritons on Metallic Nanowires. Phys. Rev. Lett..

[99] Wang W., Rosenmann D., Czaplewski D.A., Yang X., Gao J. (2017). Realizing structural color generation with aluminum plasmonic V-groove metasurface. Opt. Express.

[100] St. John A., Price C.P. (2014). Existing and Emerging Technologies for Point-of-Care Testing. Clin. Biochem. Rev..

[101] Shaw L.V. (2016). Practical challenges related to point of care testing. Pract. Lab. Med..

[102] Zarei M. (2017). Portable biosensing devices for point-of-care diagnostics: Recent developments and applications. Trends Anal. Chem..

[103] Taylor A.B., Zijlstra P. (2017). Single-Molecule plasmon Sensing: Current Status and Future Prospects. ACS Sens..

[104] Zhang Z., Chen Z., Cheng F., Zhang Y., Chen L. (2017). Highly sensitive on-site detection of glucose in human urine with naked eye based on enzymatic-like reaction mediated etching of gold nanorods. Biosens. Bioelectron..

[105] Yip P.M., Venner A.A., Shea J., Fuezery A., Huang Y., Massicote L., Tetreault N., Tomalty C., Shaw J.L.V. (2018). Point-of-care testing: A position statement from the Canadian Society of Clinical Chemists. Clin. Biochem..

[106] Rafique R., Baek S.H., Nguyen T.P., Park K.Y., Kim E.B., Kim J.G., Park J.P., Kailasa S.K., Kim H.J., Chung C. (2018). Gold-copper nanoshell dot-blot immunoassay for naked-eye sensitive detection of tuberculosis specific CFP-10 antigen. Biosens. Bioelectron..

[107] Goel S. (2018). From waste to watts in micro-devices: Review on development of Membranedand Membraneless Microfluidic Microbial Fuel Cell. Appl. Mater. Today.

[108] Mazzotta F., Höök F., Jonsson M.P. (2012). High throughput fabrication of plasmonic nanostructures in nanofluidic pores for biosensing applications. Nanotechnology.

[109] Gomez F.A. (2013). The future of microfluidic point-of-care diagnostic devices. Bioanalysis.

[110] Oh B.R., Huang N.T., Chen W., Seo J.H., Chen P., Cornell T.T., Shanley T.P., Fu J., Kurabayashi K. (2014). Integrated Nanoplasmonic Sensing for Cellular Functional Immunoanalysis Using Human Blood. ACS Nano.

[111] Cappi G., Spiga F.M., Moncada Y., Ferretti A., Beyeler M., Bianchessi M., Decosters L., Buclin T., Guiducci C. (2015). Label-Free Detection of Tobramycin in Serum by Transmission-Localized Surface Plasmon Resonance. Anal. Chem..

[112] Wang Y., Zhou J., Li J. (2017). Construction of Plasmonic Nano-Biosensor-Based Devices for Point-of-Care Testing. Small Methods.

[113] Wang L.J., Chang Y.C., Sun R., Li L. (2017). A multichannel smartphone optical biosensor for high-throughput point-of-care diagnostics. Biosens. Bioelectron..

[114] Brolo A.G. (2012). Plasmonics for future biosensors. Nat. Photonics.

[115] Kim J., Campbell A.S., Wang J. (2018). Werable non-invasive epidermal glucose sensors: A review. Talanta.

[116] Dincer C., Bruch R., Kling A., Dittrich P.S., Urban G.A. (2017). Multiplexed Point-of-Care Testing-xPOCT. Trends Biotechnol..

[117] Zhang D., Liu Q. (2016). Biosensors and bioelectronics on smartphone for portable biochemical detection. Biosens. Bioelectron..

[118] Vilela P.H., Rodrigues J.J.P.C., Solic P.S., Saleem K., Furtado V. (2019). Performance evaluation of a Fog-assisted IoT solution for e-Health applications. Future Gener. Comput. Syst..

[119] Sacha G.M., Varona P. (2013). Artificial intelligence in nanotechnology. Nanotechnology.

[120] Ma W., Cheng F., Liu Y. (2018). Deep-Learning-Enable On-Demand Design of Chiral Metamaterials. ACS Nano.

[121] Pasluosta C.F., Gassner H., Winkler J., Klucken J., Eskofier B.M. (2015). An Emerging Era in the Management of Parkinson’s Disease: Wearable Technologies and the Internet of Things. IEEE J. Biomed. Health Inform..

[122] Klímová B., Kucča K. (2019). Internet of Things in the Assesment, Diagnostics and Treatment of Parkinson’s Disease. Health Technol..

[123] Vashist S.K. (2018). Point-of-Care Diagnostics: Recent Advances and Trends. Biosensors.

[124] Boyd M., Woolley T. (2016). Point of Care Testing. Surgery.

[125] Zarei M. (2017). Advances in point-of-care technologies for molecular diagnostics. Biosens. Bioelectron..

[126] King K.R., Grazette L.P., Paltoo D.N., MCDevitt J.T., Sia S.K., Barrett P.M., Apple F.S., Gurbel P.A., Weissleder R., Leeds H. (2016). Point-of-Care Technologies for Precision Cardiovascular Care and Clinical Research. JACC Basic Transl. Sci..

[127] Nayak S., Sridhara A., Melo R., Richer L., Chee N.H., Kim J., Linder V., Steinmiller D., Sia S.K., Gomes-Solecki M. (2016). Microfluidics-based point-of-care test for serodiagnosis of Lyme Disease. Sci. Rep..

[128] Darwish N.T., Sekaran S.D., Khor S.M. (2018). Point-of-care tests: A review of advances in the emerging diagnostic tools for dengue virus infection. Sens. Actuators B.

[129] Loubier S., Moatti J.P. (2010). Economic evaluation of point-of-care diagnostic technologies for infectious diseases. Clin. Microbiol. Infect..

[130] Garcia P.J., You P., Fridley G., Mabey D., Peeling R. (2015). Point-of-care diagnostic tests for low-resource settings. The Lancet Glob. Health.

[131] Scott S.A. (2013). Clinical Pharmacogenomics: Opportunities and Challenges at Point of Care. Clin. Pharmacol. Ther..

[132] Zhao C., Liu X. (2016). A portable paper-based microfluidic platform for multiplexed electrochemical detection of human immunodeficiency virus and hepatitis C virus antibodies in serum. Microfluidics.

[133] Rebouda J., Xub G., Garretta A., Adrikoc M., Yanga Z., Tukahebwac E.M., Rowellc C., Coopera M. (2019). Paper-based microfluidics for DNA diagnostics of malaria in low resource underserved rural communities. Proc. Natl. Acad. Sci. USA.

[134] Oliveira R.A.G., Materon E.M., Melendez M.E., Carvalho A.L., Faria R.C. (2017). Disposable Microfluidic Immunoarray Device for Sensitive Breast Cancer Biomarker Detection. ACS Appl. Mater. Interfaces.

[135] Xu D., Huang X., Guo J., Ma X. (2018). Automatic smartphone-based microfluidic biosensor system at the point of care. Biosens. Bioelectron..

